# Defect Passivation and Carrier Reduction Mechanisms in Hydrogen-Doped In-Ga-Zn-O (IGZO:H) Films upon Low-Temperature Annealing for Flexible Device Applications

**DOI:** 10.3390/ma15010334

**Published:** 2022-01-03

**Authors:** Rostislav Velichko, Yusaku Magari, Mamoru Furuta

**Affiliations:** 1 Engineering Course, Kochi University of Technology, Kami, Kochi 782-8502, Japan; 2 Graduate School of Natural Science and Technology, Shimane University, Matsue, Shimane 690-8504, Japan; magari.yusaku@riko.shimane-u.ac.jp; 3 School of Environmental Science and Engineering, Kochi University of Technology, Kami, Kochi 782-8502, Japan; 4 Center for Nanotechnology, Research Institute, Kochi University of Technology, Kami, Kochi 782-8502, Japan

**Keywords:** oxide semiconductors, hydrogen in In–Ga–Zn–O, defect passivation, oxygen diffusion, low-temperature activation, flexible electronics

## Abstract

Low-temperature activation of oxide semiconductor materials such as In-Ga-Zn-O (IGZO) is a key approach for their utilization in flexible devices. We previously reported that the activation temperature can be reduced to 150 °C by hydrogen-doped IGZO (IGZO:H), demonstrating a strong potential of this approach. In this paper, we investigated the mechanism for reducing the activation temperature of the IGZO:H films. In situ Hall measurements revealed that oxygen diffusion from annealing ambient into the conventional Ar/O_2_-sputtered IGZO film was observed at >240 °C. Moreover, the temperature at which the oxygen diffusion starts into the film significantly decreased to 100 °C for the IGZO:H film deposited at hydrogen gas flow ratio (R[H_2_]) of 8%. Hard X-ray photoelectron spectroscopy indicated that the near Fermi level (E_F_) defects in the IGZO:H film after the 150 °C annealing decreased in comparison to that in the conventional IGZO film after 300 °C annealing. The oxygen diffusion into the film during annealing plays an important role for reducing oxygen vacancies and subgap states especially for near E_F_. X-ray reflectometry analysis revealed that the film density of the IGZO:H decreased with an increase in R[H_2_] which would be the possible cause for facilitating the O diffusion at low temperature.

## 1. Introduction

The material design concept of amorphous oxide semiconductors (AOS) with large electron mobility was proposed more than two decades ago [1]. Later, this concept was successfully implemented, demonstrating the strong potential of using AOS with mobility of 10 cm^2^/Vs as an alternative to hydrogenated amorphous silicon (a:Si–H), the mobility of which does not exceed 1 cm^2^/Vs [2]. These results inspired subsequent research and development of AOS such as InGaZnO (IGZO), InGaO (IGO), GaZnO (GZO), InZnO (IZO), InSnZnO (ITZO), ZnSnO (ZTO) and others for various applications [3,4,5]. These materials possess key advantages, including high electron mobility (>10 cm^2^/Vs) and high transparency, suitability for low-temperature processes which enable it to be used in flexible and transparent electronics, and industrial friendly scalability due to high uniformity and large area deposition at room temperature [6,7,8]. The pioneer representative of AOS is IGZO, and extensive research has been carried out to investigate electrical properties, generation of defects [9], and methods for improving synthesis and optimization. In addition, IGZO is the first successfully commercialized oxide semiconductor for thin-film transistors (TFTs) at the industrial level [10].

Nevertheless, the synthesis of IGZO is always accompanied by the formation of various defects. For instance, the typical defects in AOS are interstitial and weakly bonded oxygen and well-known oxygen vacancies (*V_O_*) with donor-type conductivity and which act as a carrier trap resulting in negative bias illumination stress (NBIS) [11,12,13]. Defect formation is caused both by the process parameters (base pressure, oxygen content, deposition pressure and others) and by the synthesis method itself. One of the most common synthesis techniques is a sputtering deposition in a vacuum, containing high-energy plasma species such as O ions or reflected Ar ions, after interacting with the target material. These defects affect not only the film properties but also the TFT performance. Therefore, they should be properly mitigated during post-processing in order to achieve quality films and, as a result, high-performance devices. The most common treatment method for a-IGZO is thermal annealing at 300 °C [14]. However, thermal treatment at such temperatures is not suitable for flexible devices or wearable electronic applications, which have a limited processing temperature due to the use of polymer substrate. This limitation requires the development of new approaches to achieve comparable electrical properties at a relatively low temperature. Several methods have already been proposed (these methods are referred to as “activation process of IGZO”), such as active IGZO layer oxidation at low temperatures by O_2_ wet and O_3_ annealing [15,16], high-pressure O_2_ and N_2_ gas annealing [17], hydrogen injection and oxidation for low-temperature aqueous solution-processed IGZO TFT [18], microwave and e-beam annealing [19], capacitive coupled plasma-assistant IGZO magnetron sputtering [20], and mechanochemical and thermal treatment [21]. Our research group has recently reported that adding hydrogen to the sputtering atmosphere can effectively reduce the activation temperature of IGZO films [22,23,24]. Table 1 shows the recent progress in different activation methods and the obtained TFT mobility.

Hydrogen is one of the most common impurities and can be found in all deposition apparatuses. Many experimental and theoretical studies have been conducted to better understand the role of H in OS. It is known that H results in an increased carrier density (N_e_) in ZnO and IGZO due to its shallow donor behavior [25,26]. Miyase et al. reported that the typical H content in IGZO film deposited by standard vacuum equipment is 10^20^ cm^−3^. The authors concluded favorable and unfavorable effects on TFT performance [27]. First principle calculations revealed that H upshifts the Fermi-level and therefore increases the electrical conductivity of IGZO [28]. On the other hand, the incorporation of H into the IGZO film suppresses the metal-metal (*M–M*) related defects near the conduction band minimum by forming metal-hydrogen (*M–H*) bonds [29]. Moreover, the formation of *M–H* bonds occurs by H stabilization at the *V_O_* site. The electron take-off angle dependence performed by hard X-ray photoelectron spectroscopy (HAXPES) showed the *V_O_* reduction in bulk and near-surface regions due to the H binding to the metal [30]. Thus, the addition of H can be considered as an effective method of *V_O_* reduction in IGZO film, resulting in improved device stability [31]. According to several reports, incorporating H into the film is not only limited by the IGZO synthesis process, but it should also be considered within the TFT fabrication process. It was shown that H can diffuse from the passivation layer, such as SiO*_x_* etch-stopper, or from the Al_2_O_3_ gate insulator layer to the IGZO channel, and that this diffusion leads to an improvement in TFT transfer characteristic [32,33]. Nomura et al. evaluated H diffusion into a non-hydrogenated a-IGZO film. The authors reported that H can diffuse at a depth of up to 200 nm even at relatively low annealing temperatures [34]. Despite these extensive studies into the H effects, the mechanism of low-temperature activation for H doped IGZO films remains unclear.

We previously reported that H addition led to a drastic N_e_ decrease after the annealing at a relatively low temperature of 150 °C [22]. However, an explanation of the causes leading to low-temperature activation has not been clarified yet. In this study, we focused on elucidating the mechanism causing low-temperature activation in H doped IGZO films. In situ Hall measurements performed in both air and vacuum showed the importance of the O in the annealing atmosphere to control carrier density and promote defect repair through O diffusion. Incorporation of hydrogen into the IGZO film decreased the starting temperature of O diffusion into the film with an increase in the hydrogen content in the film. The near Fermi level defects of H doped IGZO film analyzed by HAXPES decreased after annealing at 150 °C and were comparable to that of conventional IGZO film annealed at 300 °C. Finally, the X-ray reflectometry (XRR) analysis revealed a decrease in the film density after incorporation of H.

## 2. Materials and Methods

### 2.1. IGZO Film Deposition Conditions

**Figure A1:**
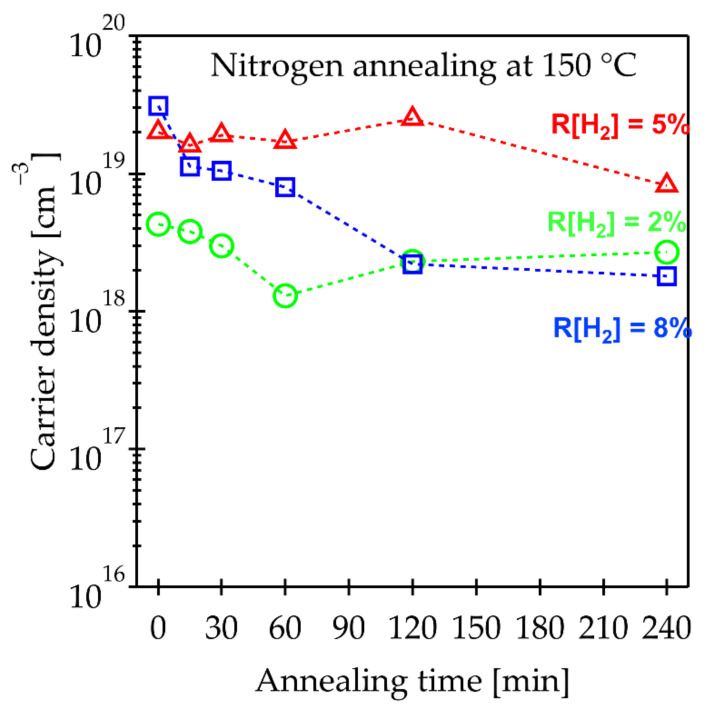
Annealing time dependence of the carrier density for H-doped IGZO films after 1 h annealing in N_2_ at 150 °C.

We deposited 50 nm thick IGZO films on a 100 mm diameter glass substrate by DC magnetron sputtering in an Ar/O_2_/H_2_ gas mixture with a total gas flow of 30 sccm using ceramic InGaZnO_4_ target (atomic ratio of In:Ga:Zn = 1:1:1) at room temperature. The O_2_ and H_2_ gas ratios were defined as R[O_2_] = O_2_/(Ar + O_2_ + H_2_) and R[H_2_] = H_2_/(Ar + O_2_ + H_2_), respectively. In order to eliminate the influence of water from the atmosphere and residual H content after deposition, the load lock and main chamber were pumped out to a back pressure below 1 × 10^−5^ Pa and 5 × 10^−5^ Pa prior to each deposition, respectively. In addition, before each deposition, the IGZO target surface was properly conditioned using a pre-sputtering process on a dummy wafer for 40 min at each R[H_2_] to achieve stable and reliable deposition environment. The R[O_2_] was fixed at 1% for both the conventional and the hydrogen-containing films, while R[H_2_] was varied from 0%–8%. The DC power, deposition pressure and distance from the target to the substrate were 80 W (0.99 W/cm^2^), 1.0 Pa and 82 mm, respectively. All films were annealed in air using a hot plate for 1 h at a temperature of 150 °C. For the annealing in N_2_ (appendix Figure A1) all films were annealed by rapid thermal annealing furnace (ADVANCE RIKO Inc., MILA-3000, Yokohama, Japan). The annealing chamber was continuously purged with N_2_ at a constant flow of 500 mL/min for 20 min in order to minimize the influence of the residual atmosphere on the carrier density. After one hour annealing, the sample was cooled to room temperature with the same N_2_ flow and only then extracted for further analysis.

### 2.2. Film Analysis Methods

The corresponding film thickness for electrical and structural properties was extracted from the ellipsometry analysis. The Cauchy model consisting of the IGZO as top layer and 0.7 mm thick glass substrate as a bottom layer was utilized. The measurements were done at three angles of incidence of 55°, 60° and 65°. The fitting was performed within 1 eV to 3 eV with backside correction. Then the calculated values were fitted until the minimum Mean Square Error (MSE) between the fit and experimental results was achieved. Obtained MSE values were between 3.7 and 4.5, which indicates that the model obtained was sufficiently accurate compared to the experimental data during the measurement process. The electrical properties were measured by the AC/DC Hall Effect Measurement system using van der Pauw geometry (TOYO Corp., ResiTest 8403, Tokyo, Japan). For routine Hall measurements, a set of four square 7 mm × 7 mm samples dimension was prepared and measured to obtain accurate and reliable results. Next, the average value of each independent set of the samples was calculated and used for analysis. In addition, the excitation current during Hall measurement was individually set up for every set of samples due to the different electrical properties of the film. Thus, it allowed the Hall voltage to be maintained at about 1 mV, and as a result kept the signal to noise ratio at the same level, additionally improving the measurement accuracy. The in situ Hall measurement program performed the following three actions. First, the sample was heated to a target temperature. Then it was allowed to stand for 5 min in order to stabilize the sample temperature, after which time the measurement was taken. These actions were performed on target temperatures in the range from 40 °C to 300 °C (250 °C for H doped IGZO) at 10 °C intervals. Before the in situ Hall measurements were taken, it was necessary to define the excitation current and AC/DC measurement mode at a specific temperature range due to continuous variation of the N_e_ during the heating in the air. Detailed parameters of the in situ Hall measurement are shown in Table A1. For the vacuum measurements, the chamber with the sample inside was properly evacuated with a rotary pump to a base pressure < 0.4 Pa. In addition, measurements were started after 60 min of waiting time to stabilize and minimize the effect of the residual atmosphere. Film density was analyzed by XRR (Rigaku Corp., SmartLab, Tokyo, Japan). Electronic structure was investigated by HAXPES with an excitation X-ray energy of 7940 eV at the BL47XU undulator beamline in SPring-8.

## 3. Results and Discussion

### 3.1. Conventional IGZO Film

**Figure 1:**
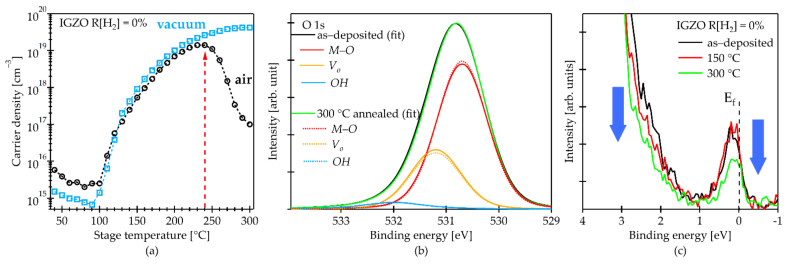
Properties of conventional IGZO film: (**a**) carrier density obtained during the in situ Hall measurement in air and vacuum; (**b**) O 1s spectra before and after 1 h annealing in air at 300 °C; (**c**) electronic structure around the band gap region for as-deposited and after annealing at various temperature.

Figure 1a shows the changes of carrier density (N_e_) in conventional IGZO film, which was deposited by sputtering in Ar/O_2_ gases, as a function of stage temperature measured by in situ Hall measurement in vacuum and ambient air. Before stage heating, the N_e_ in air was slightly higher than that in vacuum due to H_2_O absorption of the films surface, and the N_e_ did not change significantly from the initial values up to the stage temperature (T_s_) of 100 °C. When T_s_ exceeded 100 °C, the N_e_ significantly increased but exhibited almost the same values between air and vacuum annealing up to T_s_ of 230 °C. The N_e_ continuously increased for vacuum annealing, whereas N_e_ was found to decrease for air annealing as T_s_ increased more than 240 °C. The main difference between the air and vacuum measurements was observed at the T_s_ of >240 °C. This result indicates the importance of oxygen in annealing atmosphere to effectively control the carrier density. Oxygen diffusion into the film and subsequent oxidation would occur at the T_s_ of >240 °C. The Hall mobility (data not shown here) varied from 2 to 18 cm^2^/Vs depending on the N_e_, which is consistent with previous reports and explained by the percolation conduction model of carrier transport in oxide semiconductors [7,35]. 

Figure 1b shows the HAXPES O 1s core spectra of conventional IGZO film before and after air annealing at 300 °C. A slight difference in the shape of the peaks before and after annealing was found in the higher binding energy region. Three clear distinguishable Gaussian–Lorentzian curves after the O 1s spectra deconvolution were assigned to 530.7 eV, 531.2 eV and 532.0 eV, corresponding to the metal-oxygen bonds (*M–O*), oxygen vacancies (*V_O_*) and oxygen-hydrogen (*OH*), respectively [21,36,37]. Table 2 is a summary of the relative area ratio of the obtained peaks. Relative area ratio of the *V_O_* decreased after air annealing, while that of the *M–O* increased. Carrier density variation in an IGZO film is predominantly attributed to *V_O_* generation and termination in the film [11,38,39]. Therefore, the N_e_ variation observed in Figure 1a during the in situ Hall measurement suggests that the O diffusion effectively reduces the *V_O_*.

Figure 1c shows the valence band configuration from Fermi energy (E_F_). Differences in the near-valence band maximum (near-VBM) and the near-conduction band minimum (near-CBM) (i.e., near Fermi level defects) states were clearly observed. The near-VBM states decreased with an increase in annealing temperature. On the other hand, although the near E_F_ defects did not change after annealing at 150 °C, these were decreased by annealing at 300 °C. We previously reported that transfer characteristics of the TFT with conventional IGZO channel significantly improved after annealing at 300 °C in air [22]. Thus, the near E_F_ defects strongly influence the electrical properties of the IGZO TFTs. Furthermore, the required annealing temperature to obtain good electrical properties and reliability for the IGZO TFTs is typically 300 °C [14,40].

Based on the in situ Hall measurement and HAXPES analysis results, air annealing at T_s_ of >240 °C effectively promotes an active diffusion of oxygen into the film, resulting in a marked reduction of both the *V_O_* and near E_F_ defects. In other words, the oxygen diffusion into the film during annealing plays an important role for reducing *V_O_* and subgap states especially for near E_F_.

### 3.2. Hydrogen-Doped IGZO Films

#### 3.2.1. Enhanced Oxygen Diffusion at Low Temperature

**Figure 2:**
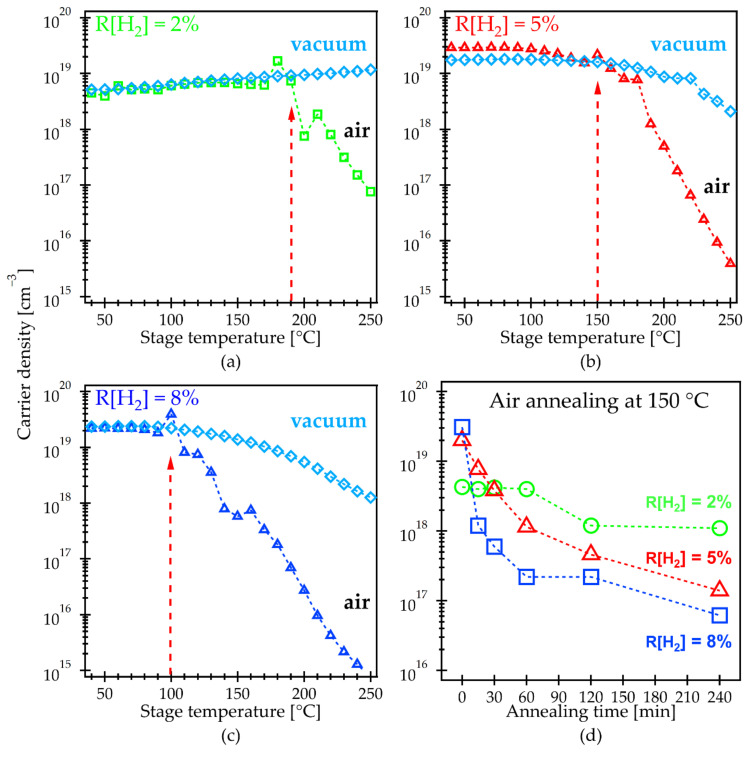
Carrier density of H doped IGZO films obtained from the in situ Hall measurement performed in air and vacuum atmosphere: (**a**) R[H_2_] = 2%; (**b**) R[H_2_] = 5%; (**c**) R[H_2_] = 8%; (**d**) Carrier density variation as a function of annealing time at fixed temperature of 150 °C performed in air.

Hereafter, we will focus on the properties of hydrogen-doped IGZO films (IGZO:H), which were deposited by sputtering in Ar/O_2_/H_2_ gases, to discuss the mechanism for low-temperature activation of the IGZO:H TFTs [22,23,24]. Figure 2 shows the in situ Hall measurement results of the N_e_ in the IGZO:H films deposited with R[H_2_] of (a) 2%, (b) 5% and (c) 8%. The N_e_ of the as-deposited IGZO:H films increased with an increase in the R[H_2_] since hydrogen acts as a shallow donor in an IGZO film. All films showed the similar N_e_ values in low-temperature region between air and vacuum annealing; however, the clear difference in the N_e_ was observed between air and vacuum annealing when T_s_ increased. The critical temperature, at which the N_e_ in air annealing started to decrease, was found at approximately 190 °C for the film with R[H_2_] of 2%. This temperature was lower than the value (240 °C) observed from conventional IGZO film as shown in Figure 1a. We also found that the critical temperature further decreased to approximately 100 °C for the film with R[H_2_] of 8%. As we discussed in Section 3.1, the critical temperature is correlated with the predominant O diffusion process in the film. Thus, in situ Hall measurement results clearly indicated that the critical temperature, at which the O diffusion starts in the film, could be reduced by hydrogen doping in the IGZO. Furthermore, the starting temperature of O diffusion into the film decreased with an increase in the hydrogen content in the film.

Figure 2d shows the annealing time dependence of the N_e_ in the IGZO:H films with different R[H_2_] values. Annealing was carried out at 150 °C in ambient air since this temperature can effectively activate the IGZO:H TFTs with good electrical properties and reliability as we previously reported [22,23,24]. A minor change in the N_e_ of about 10^18^ cm^−3^ was observed within the whole range of the annealing time for the film deposited at R[H_2_] of 2%. On the other hand, the N_e_ for the films with R[H_2_] of 5 and 8% declined sharply within the annealing time less than 60 min. Furthermore, an initial slope of the N_e_ decrease was much larger for the film with R[H_2_] of 8% than that with 5%, suggesting higher oxygen diffusion coefficient for the film with R[H_2_] of 8%. This difference in the slopes of the N_e_ decrease is consistent with the decrease in the critical temperature observed by in situ Hall measurements. We also performed the same analysis in the N_2_ atmosphere, as shown in Figure A1. The N_e_ did not change significantly throughout the annealing time and was at a relatively high level of 10^18^–10^19^ cm^−3^ for all the films with different R[H_2_] values, additionally supporting an importance of the O presence in the annealing ambient. The results of the H ratio influence on the electrical properties of IGZO and IGZO:H films have been previously published by our research group [22,41]. Hall effect mobility before and after annealing in ambient air for 1 h at a different temperature using a hot plate is shown in Table A2.

#### 3.2.2. Subgap Defects Reduction through Oxygen Diffusion

**Figure 3:**
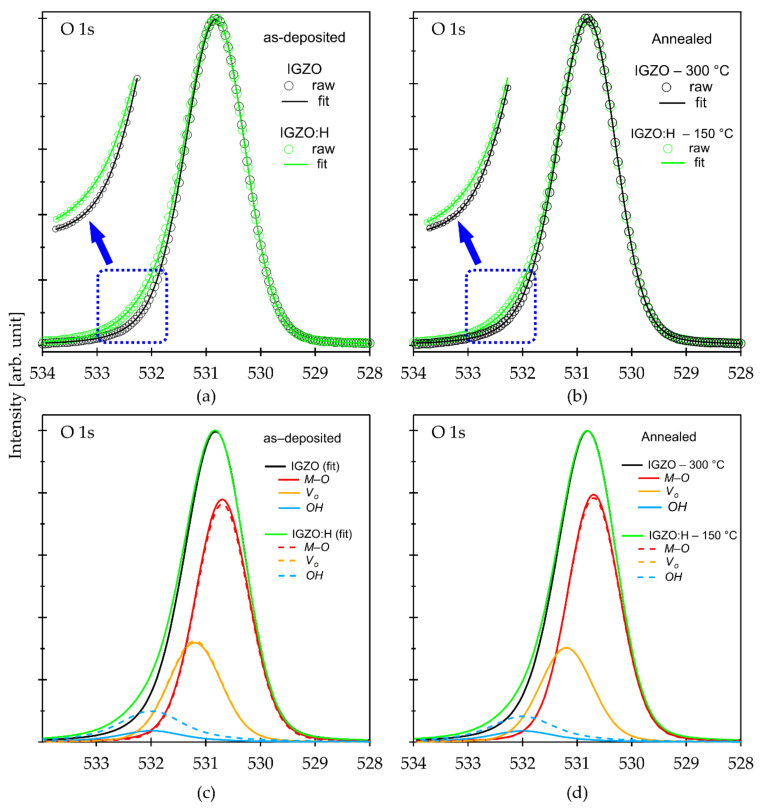
O 1s spectra of IGZO and IGZO:H films: (**a**) and (**c**) as-deposited before and after deconvolution, respectively; (**b**) and (**d**) annealed films in air for 1 h before and after deconvolution, respectively.

Next, we analyzed chemical bonding states and subgap states of the IGZO:H films. Figure 3a,b shows the HAXPES O 1s core spectra of the IGZO:H film deposited at R[H_2_] of 8% along with conventional IGZO before and after annealing, respectively. In Figure 3b, the 300 °C annealed conventional IGZO and the 150 °C annealed IGZO:H films were compared. A noticeable difference as a shoulder in the higher binding energy region was observed for the IGZO:H film both before and after annealing, as can be seen in Figure 3a,b, respectively. All peaks were similarly fitted by three Gaussian–Lorentzian curves corresponding to the *M–O*, *V_O_* and *OH*. For the as-deposited films, the relative area ratio of *OH* bonds significantly increased from 4.95% for the conventional IGZO film to 12.63% for the IGZO:H film due to an incorporation of hydrogen into the film. On the other hand, that of *V_O_* in the as-deposited IGZO:H film was 26.08%, which is slightly less than that of the conventional as-deposited IGZO film (27.35%). This suggests that the termination of the *V_O_* occurred even during the deposition process in a H-containing atmosphere. The area ratio of *V_O_* in the IGZO:H film further decreased after annealing at 150 °C to 25.08%, which is lower than that in the conventional IGZO film after annealing at 300 °C. Relative area ratios of the IGZO and IGZO:H films are summarized in Table 3. Note that the oxygen diffusion into the IGZO:H film deposited at R[H_2_] of 8% was observed at a temperature more than 100 °C as shown in Figure 2c.

**Figure 4:**
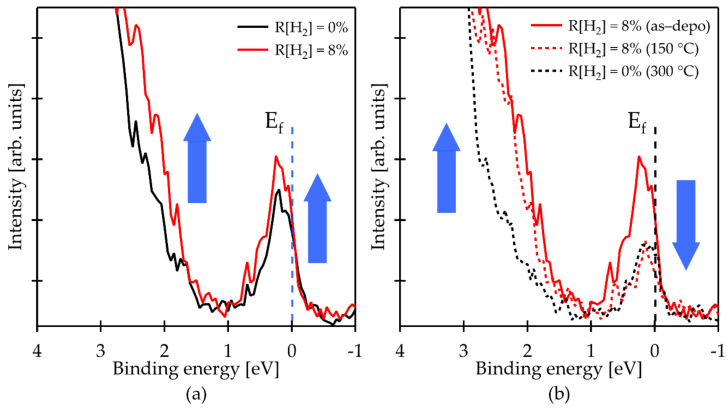
HAXPES spectra around band gap of the IGZO films deposited at different R[H_2_] and at fixed R[O_2_] = 1%: (**a**) as-deposited; (**b**) before and after annealing for 1 h in air at different temperature.

Figure 4 shows the valence band configuration from E_F_ of the (a) as-deposited and (b) annealed films, respectively. As can be seen in Figure 4a, the near-VBM states of as-deposited IGZO:H film (R[H_2_] = 8%) were markedly increased as compared with the as-deposited conventional IGZO film, whereas near-CBM states of the IGZO:H film were slightly increased. Several possible causes of the subgap states have been reported by theoretical and experimental studies [11,31,36,42,43,44,45]. Körner et al. reported that oxygen deficiencies (i.e., *V_O_*) of metal-metal (*M–M*) bonds, such as In–Zn pair defects, create defects near E_F_. By doping with hydrogen, hydrogen terminates these defects as In–Zn:H, which has an energy above the VBM within 0.4 eV (near VBM states) [29,31]. Thus, the increase in near-VBM states would be originated by the formation of *M–M:H* bonds owing to a hydrogen doping in the film.

After the annealing at 150 °C as shown in Figure 4b, the near E_F_ defects of the IGZO:H film significantly decreased to the same level as that of the conventional IGZO film after annealing at 300 °C. It should be noted that the decrease in the near E_F_ defects of the IGZO film strongly correlated with the oxygen diffusing into the film during annealing as we discussed in Section 3.1. The decrease in the near E_F_ defects of the IGZO:H was observed after annealing at 150 °C. This result can also be explained by the oxygen diffusion into the film during the annealing. The in situ Hall measurement in Figure 1a and Figure 2c revealed the starting temperature of O diffusion in the IGZO and IGZO:H (R[H_2_] = 8%) films was approximately 240 °C and 100 °C, respectively. These results demonstrate that hydrogen doping in the IGZO film reduces starting temperature of oxygen diffusion into the film; as a consequence, the near E_F_ defects can be effectively reduced by O diffusion at relatively low-temperature annealing. On the other hand, a slight decrease in the near-VBM states was also observed from the IGZO:H film after annealing at low temperature, which can be due to a termination of the deep *V_O_* by O diffusion. However, inefficient elimination of these states by O diffusion can be explained by the thermally stable *M–M:H* bonds with high binding energy [31]. Therefore, hydrogen doping is a promising way to reduce the near E_F_ defects in the IGZO film at low temperature for improving the TFT performances of flexible devices.

### 3.3. Structural Analysis of IGZO:H Films

**Figure 5:**
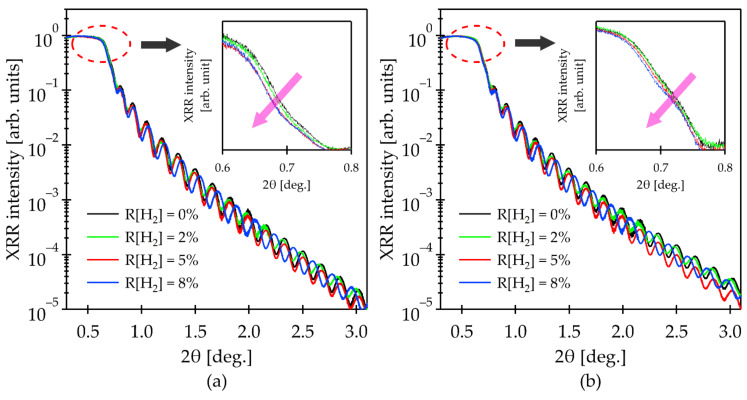
XRR results of the IGZO films deposited at different R[H_2_] and at fix R[O_2_] of 1%: (**a**) as-deposited; (**b**) after annealing in air for 1 h at the annealing temperature of 150 °C. The insets show the reflectivity intensity near the critical angle representing the change in film density upon the hydrogen incorporation.

We investigated the structural properties of IGZO and IGZO:H films by XRR analysis to elucidate the possible cause of enhanced O diffusion into the film. The evaluation of the film was done at grazing angles of incidence from 0 to 3 degrees as shown in Figure 5. The XRR results were analyzed by fitting the model with the corresponding film thickness obtained from the ellipsometry analysis to the experimental data. Thickness of the films used in this study was in the range of 45–53 nm.

Table 4 shows the extracted values of the film density after the fitting. It can be seen that H incorporation gradually decreased the initial density of the as-deposited films by 1.2%, 3.2% and 3.5%, which were deposited at R[H_2_] = 2%, 5% and 8%, respectively. We speculate that the decrease in film density observed from the IGZO:H is due to the increased content of *OH* groups as was revealed by HAXPES analysis since the film density decreased with an increase in R[H_2_]. The tendency for the decrease in film density remained after annealing at 150 °C. However, it is worth noting that although the density of the films deposited at R[H_2_] of 0 and 2% decreased from the as-deposited films after annealing at 150 °C, those at R[H_2_] of 5% and 8% increased. It was reported that structural relaxation without noticeable densification occurs at a low-temperature range (<300 °C) [46]. Thus, the film densification through low-temperature (150 °C) annealing, which was observed from the IGZO:H films deposited at R[H_2_] of 5% and 8%, would be caused by the oxygen diffusion in the films. Lastly, the rate of intensity decay at higher angle was slightly higher for IGZO:H film deposited at R[H_2_] of 5%. This is due to the difference in root-mean-square roughness (RMS) of the film which was 0.77 nm, while the RMS of the other films was in the range of 0.64–0.67 nm.

The XRR result is in good agreement with the in situ Hall measurements. Comparing the film density as listed in Table 4 and in situ Hall measurements of the N_e_ variation shown in Figure 2a–c, the higher H content showed a lower film density and the shift of the O diffusion starting temperature toward a lower temperature side. Thus, it can be considered that the H doping into the IGZO film enhanced the O diffusion into the IGZO:H film during low-temperature annealing due to a decrease in film density. As a consequence, the amount of *V_O_* and the defects near E_F_ could be reduced by oxidation of the films.

## 4. Conclusions

We investigated the mechanism for reducing the activation temperature of the IGZO:H films. In situ Hall measurements of conventional IGZO film performed in air revealed that the oxygen diffusion from the ambient air into the conventional IGZO film begins at a temperature of >240 °C, promoting subsequent oxidation, effective carrier density and near Fermi level defects reduction. Incorporation of H into the IGZO film resulted in a significant decrease in the oxygen diffusion start temperature from 240 °C to approximately 100 °C. In addition, the results of time-dependence annealing in air at 150 °C showed the decrease in the initial slope of the N_e_ decrease, suggesting higher oxygen diffusion coefficient for higher hydrogen content in the film. HAXPES results showed that the near E_F_ defects of the H doped IGZO film after low-temperature annealing at 150 °C were comparable to that of the conventional IGZO film annealed at 300 °C. This indicates that the near E_F_ defects can be effectively reduced by the enhanced O diffusion at low temperature. XRR analysis revealed structural changes upon H incorporation which resulted in a decrease in the film density, which might be the reason facilitating the O diffusion into the bulk region at low temperature. However, the hydrogen doping has a stronger effect on the change in carrier density compared to the change in film density upon low-temperature annealing. Therefore, additional studies are needed to establish the relationship between structural and electrical changes in hydrogen-doped IGZO films to further improve controllability. We believe that hydrogen introduction into the IGZO films holds promise for low-temperature applications, such as flexible electronics.

## Figures and Tables

**Table 1:** Summary of low-temperature activation methods of a-IGZO and TFT mobility.

Activation Method	Temperature (°C)	TFT Mobility (cm^2^/Vs)	References
O_2_ wet annealing	150	5.0	[15]
O_3_ annealing	≤250	11.4	[16]
High-pressure annealing in O_2_	100	10.6	[17]
Hydrogen injection and oxidation	250	3.8	[18]
Microwave and e-beam annealing	Room	8.1/11.2	[19]
Capacitive coupled plasma-assistant Magnetron sputtering	100	26.0	[20]
Mechanochemical treatment	200	12.81	[21]
Ar + O_2_ + H_2_ magnetron sputtering	150	13.4–18.9	[22,24]

**Table 2:** Relative area ratio of the metal-oxygen (*M–O)*, oxygen vacancies (*V_O_*) and oxygen-hydrogen (*OH*) of the conventional IGZO film before and after annealing in air for 1 h after deconvolution of O 1s spectra.

Temperature	*M–O (%)*	*V_O_* (%)	*OH* (%)
As-deposited	67.70	27.35	4.95
T_ann_ = 300 °C	68.63	26.42	4.95

**Table 3:** Relative area ratio of the metal-oxygen (*M–O)*, oxygen vacancies (*V_O_*) and oxygen-hydrogen (*OH*) in the IGZO and IGZO:H films before and after annealing in air for 1 h after deconvolution of O 1s spectra.

Temperature	R[H_2_] (%)	*M–O*	*V_O_*	*OH*
As-deposited	0	67.70	27.35	4.95
	8	61.28	26.08	12.63
T_ann_ = 300 °C	0	68.63	26.42	4.95
T_ann_ = 150 °C	8	64.09	25.08	10.83

**Table 4:** IGZO and IGZO:H film density (g/cm^3^) change after deposition at different R[H_2_].

R[H_2_] (%)	0	2	5	8
As-deposited	6.124	6.049	5.926	5.913
T_ann_ = 150 °C	6.075	6.035	5.985	5.922

## Data Availability

The data presented in this study are available on request from the corresponding author.

## Appendix A

**Table A1:** In situ Hall measurement parameters utilized for analysis of the conventional and hydrogen-containing IGZO films performed in air atmosphere.

R[H_2_] (%)	Temperature Range (°C)	Excitation Current	Hall Mode
0	40–110	100 nA	AC
	120–150	10 μA	
	160–200	50 μA	
	210–250	300 μA	DC
	260–300	50 μA	
2	40–60	100 μA	DC
	70–100	20 μA	
	110–150	5 μA	
	160–200	1 μA	
	210–250	500 nA	
5	40–100	1 mA	DC
	110–180	10 μA	
	190–250	300 nA	AC
8	40–90	1 mA	DC
	100–130	1 μA	AC
	140–200	20 nA	
	210–250	5 nA	

**Table A2:** Hall effect mobility (cm^2^/Vs) obtained by measurements of the IGZO and IGZO:H films before and after annealing for 1 h in ambient air at different temperature using a hot plate.

R[H_2_] (%)	0	2	5	8
As-deposited	9.6	13.0	16.0	13.0
T_ann_ = 150 °C	13.5	15.0	13.0	12.7
T_ann_ = 200 °C	17.8	13.5	12.3	11.3
